# Dental implants in immunocompromised patients: a systematic review and meta-analysis

**DOI:** 10.1186/s40729-019-0191-5

**Published:** 2019-11-28

**Authors:** Fabian Duttenhoefer, Marc Anton Fuessinger, Yasmin Beckmann, Rainer Schmelzeisen, Knut A. Groetz, Martin Boeker

**Affiliations:** 1 Department of Oral and Craniomaxillofacial Surgery, University Hospital Freiburg, Hugstetter Str. 55, 79106 Freiburg, Germany; 2 Department of Oral and Maxillofacial Surgery, HELIOS Dr. Horst-Schmidt-Kliniken, Academic Teaching Hospital University Mainz, Ludwig-Erhard-Str. 100, 65199 Wiesbaden, Germany; 3 Institute of Medical Biometry and Statistics, Medical Data Science, Faculty of Medicine and Medical Center, University of Freiburg, Freiburg, Germany

## Abstract

**Objective:**

Impaired health conditions and related lack of adequate host healing are among the most important conditions that account for dental implant failure. Today clinicians face an increasing number of immunocompromised patients requesting implant-based rehabilitation. To provide clinical evidence for prospective decision-making, the aim of this systematic review and meta-analysis was to analyse the influence of immunodeficiency on dental implant survival.

**Methods:**

The study was conducted according to the PRISMA Statement and the principles of the Cochrane Collaboration. MEDLINE and Web of Science were searched. Results were calculated by the pooled incidence of implant loss. Reported odds ratios (OR) from fully adjusted models were preferred. Distinct risk estimates were synthesised with 95% confidence intervals.

**Results:**

A total of 62 publications including 1751 endosseous implants placed in immunocompromised patients were included. For the follow-up of 24 months and longer, the mean survival rate of implants in patients with HIV was 93.1%, chemotherapy was 98.8%, autoimmune disease was 88.75%, after organ transplantation was 100%. Crohn’s disease showed a significant effect on early implant failure and resulted in increased, however not significant, implant loss.

**Conclusion:**

No significant effect of immunocompromised conditions on implant survival was detectable. Implant-based therapy in immunocompromised patients should not aggravate the general morbidity and must not interfere in life-saving therapies. A careful risk stratification prior implant therapy is fundamental. To further decipher the role of immunosuppression on dental implantology, more data from controlled and randomised studies are needed.

## Introduction

Implant-based dental rehabilitation is an expanding desire in our continuously growing and ageing society. Besides patient’s comfort and aesthetic recovery, the regeneration of the physiological function with dental implants could be directly linked to an improved overall health status and increased quality of life [1]. Still, it is undisputable that vice versa the medical status of the patient has great influence on the success rate of dental implants.

Impaired health conditions and related lack of adequate host healing are among the most important conditions that account for implant failure [2, 3].

Today, clinicians are challenged by the conflicting demands of their edentulous patients and responsible decision-making according to their patient’s medical status and history, since implant-based dental rehabilitation remains an elective treatment. Accordingly, it is mandatory to identify and exclude patients with local or systemic contraindications to ensure successful implant therapy without jeopardising patients’ health [4].

Adequate function of the immune system is a prerequisite for any non-compulsory surgery. The immune system’s inflammatory response plays a pivotal role in targeting infections as well as in orchestrating healing processes [5, 6]. Besides post-operative wound healing, the osseointegration of the inserted implant is one of the foremost steps towards successful rehabilitation [7]. It was shown that osseointegration originates from the same mechanisms as bone fracture healing and is thus directly linked to an adequate immune response [8].

However, due to a constantly improving health care with greater life expectancy as well as new indications for immunosuppressive treatments, oral and maxillofacial surgeons face an increasing number of patients that are immunocompromised or exhibit immunosuppression in their medical record. In a cross-sectional analysis regarding self-reported immunosuppression among US adults, the prevalence was 2.7, 4.2% faced immunosuppression at some time and 2.8% were under continued immunosuppression [9]. Predominantly rising in westernised societies, the prevalence of autoimmune disorders in Europe and North America had an estimated increase up to 12.5% to date [10, 11].

Transitory alterations of the immune system, for example during pregnancy or after strong allergic reactions as well as transient immunosuppression such as the open-window phenomena following intense long-duration exercise with suppressed concentration and proliferation of lymphocytes, natural killer cell activity and reduced IgA secretion in saliva are often self-limiting [12, 13]. In clinical decision-making, it is always advisable to await the recovery of the immune system prior implant therapy if possible. Besides short-term impairment of the immune system, the general process of ageing is directly linked to an increased risk of infections, malignancy, and autoimmune disorders often referred to as immunosenescence [14, 15].

The management of continuously immunocompromised patients is an immense and complex medical field and thus impossible to cover exhaustively in a general review.

Still, there is the need to address the option for implant therapy in the increasing population of patients under immunosuppressive therapy that prevents or mitigates common chronic diseases, treats HIV and cancer, or after transplantation. Given the broad range of indications for glucocorticoid therapy such as asthma, rheumatism, and connective tissue disorders, it is almost certain to encounter a patient under corticosteroid therapy who requests implant-based rehabilitation [16].

Consequently, the aim of this systematic review and meta-analysis was to compare treatment results of implant dentistry in immunocompromised patients with them in not immunocompromised patients. Particular attention was given to the cause of immunosuppression and the subsequent influence on implant survival to provide clinical evidence for prospective therapy decision-making.

## Materials and methods

### Databases and search strategy

The central question of this study was designed after the PICOS template: “In patients after a therapy with dental implants (P) and suffering from different conditions of immunosuppression/immunodeficiency (I) can there be an increased risk of implant loss observed (O) compared with non-immunosuppressed patients (C), retro- and prospective clinical interventional studies, surveillance studies, cross-sectional studies, cohort studies and case series were included (S).” The aim was to answer this question by a systematic literature search based on the PRISMA Statement.

**Fig. 1:**
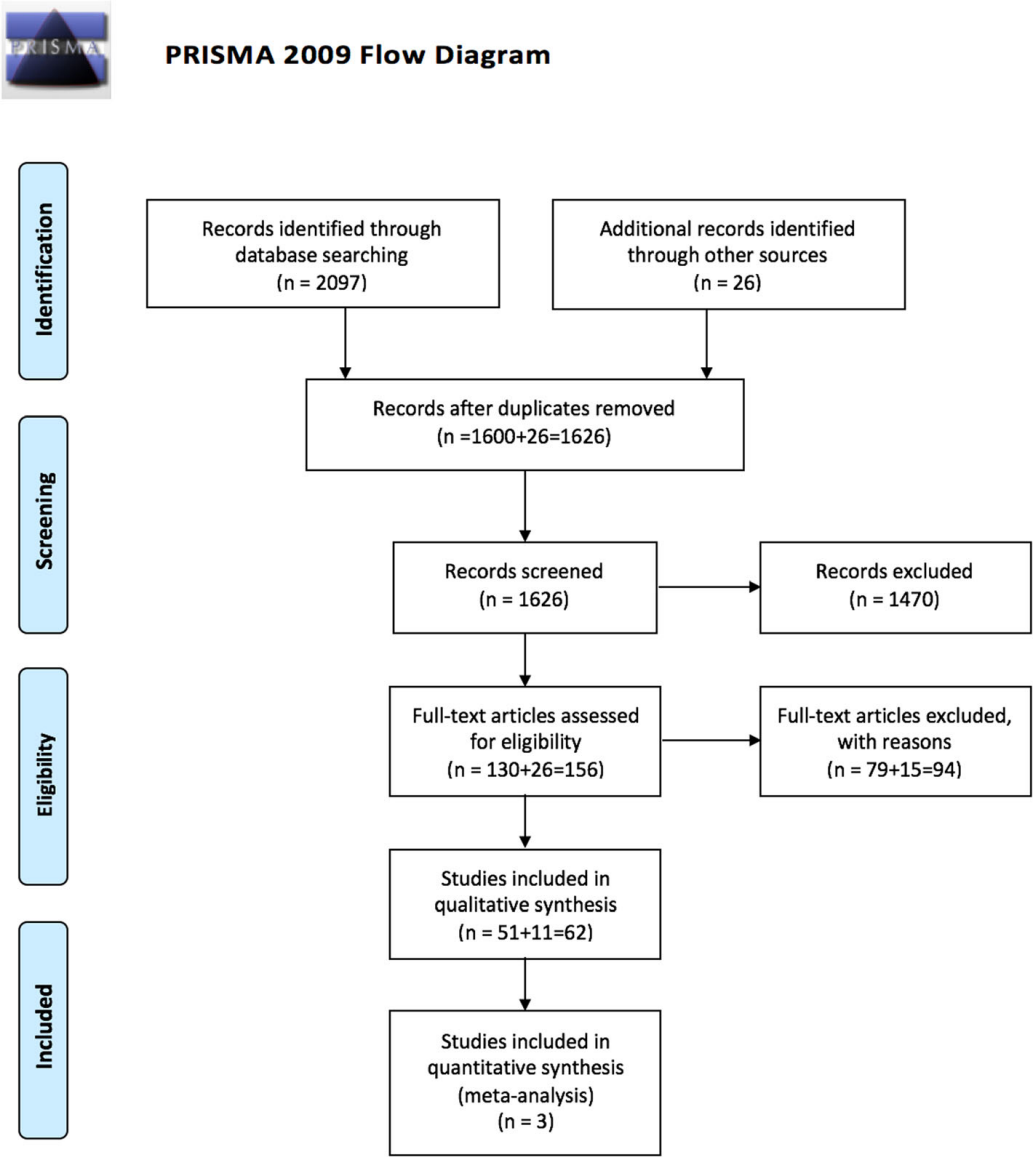
Prisma flow diagram

Hence, this systematic review and meta-analysis was conducted according to the Preferred Reporting Items for Systematic Reviews and Meta-Analyses (PRISMA) Statement [17] and the principles of the Cochrane Collaboration (Fig. 1) [18–20]. All steps were documented in a protocol prior to execution (not published). The conducted search strategy was created in close collaboration with an information scientist and used the following databases: MEDLINE via the search interface OvidSP (keyword search) and Web of Science by Thompsen (all core databases, i.e. Science Citation Index, MEDLINE, Biosis). For the detailed search history see Additional file 1. The search was conducted in November 2017.

### Inclusion and exclusion of studies

In order to limit the recorded entries to the relevant material, inclusion and exclusion criteria were specified in advance by the reviewers. The initial criteria were not subjected to any alterations throughout the study:

The following inclusion criteria were applied:

1. English or German language
2. Human - retrospective and prospective clinical interventional and surveillance studies; cross-sectional studies, cohort studies and case series

The following criteria lead to exclusion:

1. In vitro studies
2. Animal studies

Study selection and data extraction were performed by three independent reviewers. Ambiguities were discussed among the authors until consensus on inclusion/exclusion was reached.

### Data extraction

Information was extracted from each included study of the following data items and if applicable with the indicated values in brackets:

(1) author, (2) publishing year, (3) study type, (4) medical condition (HIV infection, chemotherapy, transplantation, autoimmune disease, oral cancer/squamous cell carcinoma), (5) number of patients included in the study, (6) fraction of female and male patients, (7) age of the patients (median), (8) absolute number of placed implants, (9) absolute number of failed implants, (10) time of implantation after extraction, (11) time of loading, (11) maximum follow-up period, (12) survival rate of implants, (13) localisation (mandible, maxilla), (14) generic therapy term (antiretroviral therapy, steroid containing medication, chemotherapy, immunosuppressive drugs), (15) applied drug, (16) duration of the applied therapy at the time of implantation, (17) underlying disease (Crohn’s disease, oral lichen planus, rheumatoid arthritis, scleroderma, Sjogren syndrome, dermato myositis, pemphigus vulgaris, polymyalgia rheumatica, systemic lupus erythematosus, oral cancer/squamous cell carcinoma), (18) CD4 cell count, (19) viral load, (20) prescribed antibiotic drug, (21) period in which the study was carried out, (22) type of effect estimate (relative risk, odds ratio, attributable risk/excess risk, arcsine difference, standardised mean difference, weighted mean difference, hazard ratio), (23) value of effect estimate, (24) risk of bias assessment, (25) overall objectives of the study, (26) setting and/or place of study, (27) additional information. The authors of one study were contacted by mail to clarify missing, insufficient, inadequate, or controversial data. Studies with continuous unclear or incomplete data were excluded. All data are available in the Additional file 2.

### Risk of bias assessment within the studies

All controlled studies were assessed for their risk of bias according to the tool of the Cochrane Collaboration adjusted to non-randomised studies [21]. The classifications were “low risk,” “high risk,” and “undetectable risk” of bias. Seven different categories were evaluated, including “random sequence generation,” “allocation concealment,” “blinding of participants and personnel,” “blinding of outcome assessment,” “incomplete outcome data,” “selective reporting,” and “other sources of bias.”

Results of the risk of bias assessment are reported for each individual study Table 1.

**Table 1:** Risk of bias assessment

Author	Year	Rand_seq_gen	All_conceal	Blind_per	Blind_out	Inc_data	Sel_report	Other_sources
Alsaadi	2007	Unclear	Low	Low	Low	Unclear	Low	Low
Alsaadi	2008	Low	Low	Low	Low	Unclear	Low	Low
Alsaadi	2008	Unclear	Unclear	Low	Low	Low	Low	Low
Gastaldi	2017	High	High	High	High	Low	Low	Low
Gay-Escoda	2016	High	High	High	High	Unclear	Unclear	Low
Gherlone	2016	High	Unclear	High	High	Low	Low	Low
Gherlone	2016	High	Unclear	High	High	Low	Low	Low
Kovacs	2001	High	High	High	High	Low	Low	Unclear
Krennmair	2010	High	High	High	High	Low	High	Unclear
Malo	2016	High	High	High	High	Low	Unclear	Unclear
May	2016	High	High	High	High	Low	Low	Low
Montebugnoli	2012	High	High	Low	Low	Low	Low	Low
Montebugnoli	2015	High	High	High	Low	Unclear	Unclear	Low
Moy	2005	High	High	High	Low	Unclear	Low	Low
Oliveira	2011	High	High	Low	Low	Low	Low	Low
Stevenson	2007	High	Unclear	Low	Low	Low	Low	Low
van Steenberghe	2002	High	High	High	High	Low	Unclear	Unclear
Weinlander	2010	Low	Low	Low	Low	Low	Low	Low
Westhoff	2012	High	Unclear	High	High	Low	Low	Low
Ihara	1998	High	High	High	High	Low	Unclear	Unclear

The risk of bias assessment was reported for each individual study and detected random sequence generation (Rand_seq_gen), allocation concealment (All_conceal), blinding of participants and personnel (Blind_per), blinding of outcome assessment (Blind_out), incomplete outcome data (Inc_data), selective reporting (Sel-report), and other sources of bias (Other_sources). The risk of bias was characterised as low risk, high risk, and undetectable risk (low, high, unclear).

### Qualitative synthesis

Results were reported group-wise for the following medical conditions as extracted: HIV infection, chemotherapy, transplantation, autoimmune disease, oral cancer/squamous cell carcinoma.

### Statistical analysis

Means of survival rates were calculated for medical condition as extracted (see above) from the mean survival rates of individual studies weighted for included patients. Means were calculated for studies with follow-up shorter than 6 months and longer than 24 months.

We employed the generic inverse variance method by using a random effects model to calculate the pooled incidence of implant loss [22]. Where available, the reported odds ratios (OR) from fully adjusted models were preferred. Distinct risk estimates were synthesised with 95% confidence intervals [22]. “R” was used for the conduction of all analysis and graphics (version 3.5.0) [23, 24].

## Results

A total of 2097 records were identified through the electronic database search. Twenty-six articles were found by manual search. A total of 1626 duplicate adjusted citations were selected. Of these, 1470 records were excluded after reviewing the abstracts because these papers did not meet the inclusion criteria. Of the remaining 156 citations, the full texts were examined in more detail. An additional 94 articles did not meet the inclusion criteria and were excluded. Sixty-two studies met the inclusion criteria and were included in the systematic review.

A total of 3 publications on implant therapy in immunosuppressed patients suffering from Morbus Crohn were eligible for meta-analysis (Fig. 1).

### HIV and AIDS

A total of 16 studies with 578 implants were identified regarding implant-based rehabilitation in HIV and AIDS patients. In the four prospective studies, with one controlled study, none of the authors found significant correlations between HIV-related immunodeficiency and implant failure [25–29]. The highest survival rate of 100% was seen in the only controlled prospective study after a follow-up of 6 months, with no detectable difference between HIV-infected and uninfected patients regarding clinical response and osseointegration of the implants [25]. The lowest detected survival rate among the prospective studies was 90.91% with the longest follow-up of 60 months [26]. It is important to note that this was the only study included in this review which analysed implants placed exclusively in patients meeting the AIDS criteria according to the American Center for Disease Control and Prevention with a cluster of differentiation 4 (CD4) count below 200 cells per microliter [26]. If information was available at all, the remaining studies enrolled only individuals with a stable disease based on CD4 count and HIV viral load as well as the absence of severe immunodeficiency with high recurrence of opportunistic infections, wasting disease, and disseminated malignancy in implant therapy.

In the retrospective overview study by Malo et al. 2016 analysing long-term results of dental implants in patients with systemic disorders, five HIV-positive patients were enrolled of which 4 suffered from concurrent conditions such as smoking or cardiovascular disease. Of the 40 inserted implants, 2 failed due to unknown reasons over the follow-up of up to 9 years [30]. Similar results were seen in the retrospective study by Gay-Escoda et al. 2016 where nine patients received a total of 57 implants and a survival rate of 98.25% over 78-month follow-up [31]. Out of nine case reports from 1998 to 2016 and one controlled pilot study, there was no correlation between CD4 count (CD4 counts ranging from 150 to 1000 cells/μl) and implant failure [32–37]. The success rate in all but one case reports was 100% with follow-up times ranging from 6 to 122 months. Only one out of four implants was lost in one case after 4 months due to failed osseointegration, but could be replaced successfully [38]. The detected causes of implant failure were peri-implantitis, tilted implants, failed osseointegration, and early infection.

For follow-up of 24 months and longer, the mean survival rate of implants in patients with HIV was 93.1% with an average follow-up of 31.7 months (*n* = 200, Table 2).

**Table 2:** Pooled survival rates and medical conditions

Type	*n*	FU [mo]	Survival [%]
HIV/AIDS	200	31.7	93.1
Autoimmune disease	100	72.6	88.8
Chemotherapy	51	112.5	98.8
Organ transplantation	16	47.8	100.0

Mean of survival rates and follow-up (FU) for given groups of medical condition sorted by number of included patients. Only studies with follow-up longer than 24 months are included.

### Chemotherapy

Eight studies comprising a total of 144 implants were enrolled to show the influence of chemotherapy on the success rates of dental implants. The four retrospective studies [39–41] of which only one was controlled [42] and two prospective studies [43, 44] revealed no significant influence of chemotherapy on the success rate of dental implants. In the only available controlled study performed by Kovacs et al. 2001, there was no significant difference in the Kaplan-Meier life-table analysis (Log-Rank *P* = 0.2230) on cumulative survival of dental implants comparing groups with and without chemotherapy over a time period of up to 120 months. The groups comprised 17 patients receiving 54 implants in the control group and 30 patients receiving a total of 106 implants in the group that received chemotherapy (cisplatin or carboplatin and 5-fluorouracil). The function of prosthesis was comparable in both groups with a mean prosthetic loading of the implants of around 36 months (chemotherapy 35.8 ± 26.3 months, control 36.2 ± 29.7 months). The uncontrolled prospective and retrospective studies analysed implant success and associated risk factors in the patients’ medical records of which chemotherapy was one. The study by Moy et al. 2005 analysed 10 implants placed in 10 patients undergoing chemotherapy. One implant was lost due to unknown cause resulting in a success rate of 90% (RR = 63). There was neither information given on the applied type of chemotherapy nor the follow-up time [40]. In the case reports as well as the two studies by Alsaadi et al. 2007, 2008 and the retrospective study by Ihara et al. 1998, a total of 28 implants were inserted and showed no adverse effect of chemotherapy. The success rates were 100% in all studies with follow-up times between 3 and 28 months [39, 41, 43, 45, 46].

For follow-up of 24 months and longer, the mean survival rate of implants in patients after chemotherapy was 98.8% with an average follow-up of 112.5 months (*n* = 51, Table 2).

### Immunosuppression in autoimmune diseases

A total of 19 studies, 11 case reports, 2 prospective, and 6 retrospective studies investigated the influence of autoimmune diseases and treatment modalities with steroid derivatives and immunosuppressant drugs on the survival rates of dental implants. A total of 596 inserted implants in 129 patients were examined between 6- and 156-month follow-up. Out of the patient collective, 68 were female and 1 male and in 60 cases, data on the sex of the patient was not available. In 13 investigations, the predominantly used drug to treat the underlying autoimmune disease was some sort of steroid medication [47–59]. One study applied an additional immunosuppressive drug [60]. Five studies did not mention the type of medication [30, 39, 43, 44, 61] and two studies reported the application of steroids but did not inform about the underlying autoimmune disease [39, 61]. In five studies, 9 patients that suffered from co-existing autoimmune disorders (rheumatoid arthritis and Sjogren’s syndrome, rheumatoid arthritis and dermato myositis, oral lichen planus and Sjogren’s syndrome) received a total of 39 implants of which none was lost over a follow-up time ranging from 21 to 156 months [51, 54, 55, 58, 59]. In neither of the studies did the comorbidity of autoimmune diseases affect the implant survival. The study by Malo et al. 2016 showed a survival rate of 86.1% between year one and 7 and 80% in the following 2 years. Between 9 and 11 years, the survival rate dropped to 72%. There was no detailed information on the type of disease or the therapy targeting the rheumatological disorder of the patients. The dropout rate was estimated at 12% [30].

In the reviewed case reports comprising the autoimmune diseases polymyalgia rheumatica, pemphigus vulgaris, scleroderma, Sjogren’s syndrome, and systemic lupus erythematosus, the survival rate was 100% during follow-up periods ranging from 4 to 13 years. All patients received prednisone or cortisone therapy and no effect on implant survival of either the medication or the underlying autoimmune disease was detected.

For follow-up of 24 months and longer, the mean survival rate of implants in patients with autoimmune disease was 88.75% with an average follow-up of 72.6 months (*n* = 100, Table 2).

### Rheumatoid arthritis

Three retrospective, one prospective, and four case reports analysed a total of 236 implants placed in 56 patients suffering from rheumatoid arthritis [39, 50, 51, 54, 58–61]. Seven patients were additionally diagnosed with Sjogren’s syndrome and one patient with dermato myositis [51, 54, 58, 59]. In the retrospective analysis, survival rates were 100%. Weinlaender et al. 2010 showed a survival rate of 100% of 83 implants inserted in 21 patients after a follow-up of 46 months [51]. Likewise, Krennmair et al. 2010 had no implant loss after 47.6-month follow-up in 25 patients that received a total of 95 implants [50]. In both studies, the patients received some form of corticosteroid therapy. Alsaadi et al. 2008 showed that no implant was lost in a study comprising 6 patients with 28 implants over a period of 24 months [39]. The only prospective study reported a survival rate of 92.9% with one out of 14 implants lost due to unknown reason [61]. No influence of rheumatoid arthritis on the overall survival rate of dental implants was detected.

### Morbus Crohn

Four studies were included investigating the influence of Crohn’s disease. Two studies were prospective, published in 2002 and 2008, and two studies were conducted retrospectively, published in 2007 and 2008 [39, 43, 44, 61].

The highest survival rate of over 90% was found in a recent prospective study from 2008. Herein, the influence of systemic and local factors on the occurrence of early failures was investigated. Only one implant of a total of twelve implants with modified oxidised titanium surfaces was lost during an observation period of 6 months. Still, the Crohn’s disease is significantly related to early implant failure, as described by a GEE value of 0.02 and a fisher value of 0.21 [39]. However, the information on the exact number of patients is missing. In the prospective study by van Steenberghe et al. 2002, two of the three patients lost overall three out of ten implants according to an early implant failure up to abutment connection [44]. However, these patients presented also other risk factors in addition to the existing Crohn’s disease, among them claustrophobia, poor bone quality, and smoking more than ten cigarettes a day. A comparable survival rate is shown by a retrospective study with the longest observation period of 2 years. In addition to the early implant failure, the late failure after loading was investigated. During the 2-year period, three out of twelve implants were lost [39]. Crohn’s disease resulted in increased, however not significant, implant loss (OR = 10.09; 95% CI, [0.73, 139.79]; *P* value, 0.09).

In the other retrospective study, patient data from1982 to 2003 were evaluated with regard to early implant failure associated with local and systemic risk factors [61]. A significant effect of Crohn’s disease on early implant failure was found (OR = 7.95; 95% CI, [3.47, 18.24]; *P* value, 0.001). The exact patient data, such as number of treated patients, as well as the exact number of placed and lost implants, was not provided.

**Fig. 2:**
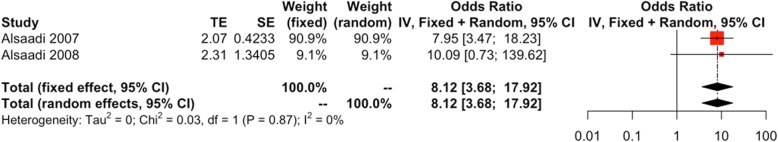
Meta-analysis of two studies investigating the effect of Crohn’s disease on early implant failure

The aforementioned studies could be included in the only meta-analysis of this systematic review. The significant pooled OR is 8.12 with a 95% CI of [3.68, 17.92] (Fig. 2).

There is a broad heterogeneity of available patient and implant information, with often missing information. Still, the investigated studies show increased implant loss in the presence of Crohn’s disease with a trend towards early implant failure.

### Immunosuppression after organ transplantation

Dealing with implant-based rehabilitation and immunosuppression after organ transplantation, a total of 6 studies with 107 placed implants in 39 patients were included of which 2 were prospective studies without control and 4 case reports. The two prospective studies were published in 2012 and 2015 [62, 63]. The case reports were published in 2004, 2011, and 2014 [64–67]. The authors found no significant correlations between immunosuppression after organ transplantation and implant failure. All identified studies and case reports describe an implant survival rate of 100% with a follow-up of 58 months as mean (3–118 months) with no effect on clinical findings or on osseointegration of the implants. The loading time after implantation differs from 3 months for the maxilla up to 9 months of the mandible. In the prospective studies, 12 months after extraction of teeth implants were placed. Regarding the case reports, it differs from 1 to 5 months as waiting time between extraction and implant placement. As immunosuppressive drugs tacrolimus, sirolimus, cyclosporine, and mycophenolate were used, steroid prednisolone was applied. The indication for immunosuppression was a liver transplantation in 26 patients and a heart transplantation in 13 patients. Twenty-seven patients receive a prophylactic antibiotic therapy, like moxifloxacin, for 6 days or amoxicillin with clavulanic acid for 5–6 days.

For follow-up of 24 months and longer, the mean survival rate of implants in patients after organ transplantation was 100% with an average follow-up of 47.8 months (*n* = 16, Table 2).

## Discussion

To date, scientific evidence regarding the influence of primary and secondary immunodeficiency or immunosuppressive treatment modalities on survival rates of endosseous implants is yet vague and very heterogeneous. Thus, evidence-based decision-making whether or not to bestow implant-based dental rehabilitation upon immunocompromised patients is still impaired. To address this issue, the present systematic review analysed the available literature on the influence of different types of immunosuppression (HIV, chemotherapy, autoimmune disorders and steroid medication, organ transplantation) on the outcome of the applied implant-based therapies in terms of implant survival.

### HIV

HIV infection and subsequently AIDS as an epidemic with devastating debilitation of the patients evolved over the past 30 years into a stable yet chronic disease. Accordingly, there is a constantly increasing number of patients in different stages of disease that are requesting implant-based dental rehabilitation. Regarding HIV-seropositive patients with a CD4 count over 200 cells/μl, the present review did not find a significant failure rate compared with healthy patients. Several studies regarding healing response, general implant infection rates, or post-operative complications linked to immunological values could not show a significant difference to healthy patients [68–70].

Moreover, only few studies focused on patients in a severely immunocompromised state with a CD4 count lower than 200 cells/μl. These patients are prone to develop symptoms of AIDS, such as opportunistic infections and neoplasia. Still, there was no evidence regarding a direct relationship between the CD4 count and the risk of post-operative infections [25–27]. In a systematic overview by Ata-Ali et al. 2015, the administration of antibiotics was deciphered as among the main influencing factors when analysing the osseointegration of dental implants in HIV-positive patients [71]. Likewise, in 75% of the analysed studies, different forms of antibiotic treatments were applied upon implant surgery. The prophylactic application of antibiotics was shown to reduce the risk of implant failure, however not influencing the risk of post-operative infections [72, 73]. Accordingly, it is advisable to consider its application when treating HIV-seropositive patients.

### Chemotherapy

Chemotherapy is one of the fundamental pillars of cancer treatment. To date, a broad variety of antineoplastic drugs designed to target certain forms of cancer are available and their number is constantly increasing. Accordingly, it is impossible to comprehensively cover all mechanisms of biological actions of chemotherapies that may interfere with implant surgery.

One of the foremost parameters of successful implant therapy, osseointegration, is not well elaborated with regard to chemotherapy. Although several animal studies have shown a negative effect on bone remodelling and fracture healing, information in humans are sparse [74, 75]. There is evidence that chemotherapy treatment may entail a multitude of negative effects on patients with pre-existing implants, such as mucositis and painful peri-implant infections as well as systemic effects such as fever and septicaemia. However, most of these reported side effects were seen on subperiosteal and blade implants, surpassed to date [76]. Contrary, it was shown that osseointegration is not necessarily impaired during the course of chemotherapy. In the case report of Steiner et al. 1995, the uncovering of two dental implants was performed uneventfully during chemotherapy to treat a non-Hodgkin’s lymphoma. Sager et al. 1990 successfully inserted a total of four implants during the recovery period of melphalan chemotherapy cycles targeting a multiple myeloma. During the follow-up of 18 months with functional prosthesis, no implants were lost and no signs of peri-implant inflammation were seen [46]. Both authors were performing surgeries with regard to the patients’ white blood cell count and emphasised the importance of a close cooperation with the oncologists. In both case reports, the patients were set under antibiotic treatment until suture removal. In the only available controlled study by Kovacs at al. 2001 on patients with squamous cell carcinoma treated either with chemotherapy (cisplatin or carboplatin and 5-fluorouracil) or ablative surgery only, there was no statistically difference on implant survival. Patients receiving chemotherapy were subjected to implant surgery around 10 months after the last cycle. Implant success rates were over 98% in both groups during a follow-up of up to 120 months. The only available meta-analyses stated that implant failure rates were not affected by chemotherapy application (risk ratio 1.02, 95% confidence interval 0.56–1.85; *P* = 0.95); however, the performed statistic was substantially affected by the inconsistency of study designs [77].

Since chemotherapy is mostly administered in cycles, with alternating treatment and recovery periods, it seems advisable to await the completion of treatment before implant therapy. Moreover, an interdisciplinary approach in close cooperation with the responsible oncologists is preferable.

### Autoimmune diseases

The breakdown of immunologic tolerance towards self-molecules leads to an immune response that subsequently manifests in several forms of autoimmune diseases [78].

The initiation factors that start this autoimmune response are mostly unknown; still there is evidence that socioeconomic, genetic, and environmental factors as well as certain types of infections play a pivotal role. Over the last decade, there is increasing evidence for a steady rise in the frequency of autoimmune diseases [11]. Approximations on the prevalence of autoimmune disorders range from 3% in Europe and North America at the beginning of the century up to 12.5% to date [10, 11]. Autoimmune disorders are predominantly rising in westernised societies, those having access to hygiene and advanced health care [11]. Consequently, there is an increasing number of patients with autoimmune disorders requesting dental implants.

The present study investigated a total of 596 inserted implants in 129 patients. With regard that information on the sex was not always available, the given information revealed a clear trend towards female patients that comprised 98% of the patients collective. It is known that autoimmune disorders predominantly occur in women. Today 75% of those suffering from autoimmune disorders are female [10].

Clinical investigations on the associations among autoimmune diseases revealed that these conditions, conventionally considered as distinct disorders, often co-exist in one patient. Typically patients are associated with an index disease and the occurrence of other autoimmune disorders is assessed within the index population [79]. Weng et al. found among others a significantly increased occurrence of rheumatoid arthritis and Sjogren disease [80]. This constellation of comorbidity was the most frequent in the assessed literature where 35 implants were inserted in 7 patients suffering from the burden of rheumatoid arthritis and Sjogren’s syndrome. In the follow-up period ranging from 46 to 156 months, no implant was lost. In addition, no other constellation of co-existing autoimmune disorders showed a significant influence on implant survival.

It is well documented that exogenous corticosteroids, including low-dose inhaled drugs, can suppress counterregulatory immune response to illness and tissue injury.

Similar mechanisms are described in response to surgery and trauma where plasma ACTH and cortisol concentrations increase. Still there is a general patient-to-patient variability with regard to age, sex, and other influencing co-factors such as anaesthesia, opioids, or infections [16]. In the analysed studies, the predominant medication in patients suffering from autoimmune disorders is prednisone or cortisone therapy. In neither of the studies, a direct effect on implant survival of either the medication was detected.

### Morbus Crohn

Crohn’s disease is a chronic inflammatory bowel disease, which mainly affects the gastrointestinal tract. Antigen-antibody complexes lead to autoimmune inflammatory reactions which is why immunosuppressive and anti-inflammatory drugs are part of the spectrum of therapy. Examining early implant failure, all three studies [43, 44, 61] concluded that Crohn’s disease is related to early implant failure. The studies of Alsaadi et al. from 2008 and 2007 substantiated this outcome statistically with significant *P* values of less than 0.001 in the study of 2007 and 0.02 in the study of 2008. The causes for the cumulative incidence of early implant failure are widely discussed. One theory implies that antigen-antibody complexes lead to autoimmune reactions in the area of the bone-implant contact, thus influencing the osseointegration of the implants [81]. Furthermore, the malnutrition, which often occurs in the course of Crohn’s disease, possibly results in a deficient bone healing around dental implants [82].

Only one study considered the late loss of implants [39]. The total observation period was 2 years. The *P* value of 0.09 is slightly above the significant value of 0.05. Yet, further studies are needed to examine more closely the late implant loss and to permit a general statement.

In the study by van Steenberghe from 2002, patients showed other medical factors additionally to the existing Crohn’s disease, including claustrophobia, poor bone quality, and smoking. Also examining these factors, the authors concluded that patients suffering from claustrophobia had an increased failure rate. Likewise, the other two factors, poor bone quality and heavy smoking, led to a higher incidence of implant failure. Still, such heterogeneous and superficial results should be considered with caution [44].

The present study indicates a clear trend towards early implant failure, which, however, needs to be proven yet in larger scale controlled trials. With regard to late implant failure, further randomised controlled studies with longer observation periods are necessary to allow for final recommendations.

### Immunosuppression after organ transplantation

Based on the fact that there is a growing number of solid organ transplant recipients, the dentists come face to face with the needs of dental rehabilitation of patients which endure a massive dental clearance before transplantation to decrease the rate of infectious diseases [83, 84]. Nevertheless, in 50% of the analysed studies, a prophylactic antibiotic treatment was administered [63, 64, 67]. Respectively, the application of antibiotics in case of dental implant placement could be advisable.

Different post-transplantation protocols prefer different immunosuppression protocol. Taking into account the results of the presented review, the choice of immunosuppressive drugs had no detectable effect on the implant survival rate. Neither steroids nor immunomodulatory drugs increase the risk of implant failure. Negative aspects of the implant site connected to immunosuppression were not seen. To conclude, the evidence of included studies is limited due to the fact of missing randomised controlled trials, but regarding the results of the named studies, which are mainly case reports, no limitation for implantation of dental implants could be seen.

### Limitations

The available studies rely on a small yet very heterogenous patient collective and are predominantly characterised by the lack of control groups and a low level of specificity. No randomised controlled studies on immunosuppression and dental implant rehabilitation were available. Further limitations were seen in the different methodological approaches of the analysed prospective and retrospective studies. Only few studies had a control group, usually the studies compared with healthy patients or provided no control group at all. Results were often summed up and not clearly identified or patient related, i.e. implant loss per patient. Furthermore, the exclusion of implant-related diseases such as peri-implantitis may be considered as a limitation. It remains unknown whether these factors are influenced by immunosuppression and may lead to implant failure at a time-point that exceeds the follow-up of the patient. Only studies in English or German language were considered accordingly data maybe missing. Such limited quality of primary studies leads to the urgent need for randomised controlled studies with more patients on the effects of immunosuppression on dental implants.

## Conclusion

To our knowledge, this is the first systematic review which collected evidence for the influence of different immunodeficient conditions (HIV/AIDS, organ transplantation, autoimmune disease, and Morbus Crohn) on the survival of dental implants. The review was conducted according to the high standards of the Cochrane Collaboration and the PRISMA statement.

There is only little evidence for deteriorating influences of immunodeficient conditions on the survival of dental implants. Regarding the analysed immunosuppressive conditions, only Crohn’s disease showed a significant effect on early implant failure and resulted in increased, however not significant, implant loss. There was no significant effect on implant survival in the remaining immunocompromised conditions detectable. However, the methodical quality of the included studies is generally low and the number of patients analysed is small. Therefore, these results should be interpreted with caution due to the low strength of evidence in case series as well as prospective and retrospective studies.

Hence, there is no data-based final conclusion on whether or not to place dental implants in patients receiving immunosuppression or suffering from immunosuppressive conditions. Implant-based therapy in immunocompromised patients should not aggravate the general morbidity and must not interfere in life-saving therapies and thus jeopardising patient’s health. In general, a careful risk stratification prior implant therapy based on the patients’ medical record and current medical status is fundamental.

## Supplementary information

**Additional file 1.** Detailed search history. The conducted search strategy used the databases: MEDLINE via the search interface OvidSP (keyword search) and Web of Science by Thompsen (all core databases: Science Citation Index, MEDLINE, Biosis).

**Additional file 2.** Detailed results table. (1) author, (2) publishing year, (3) study type, (4) medical condition (HIV infection, chemotherapy, transplantation, autoimmune disease, oral cancer / squamous cell carcinoma), (5) number of patients included in the study, (6) fraction of female and male patients, (7) age of the patients (median), (8) absolute number of placed implants, (9) absolute number of failed implants, (10) time of implantation after extraction, (11) time of loading, (11) maximum follow-up period, (12) survival rate of implants, (13) localization (mandible, maxilla), (14) generic therapy term (antiretroviral therapy, steroid containing medication, chemotherapy, immunosuppressive drugs), (15) applied drug, (16) duration of the applied therapy at the time of implantation, (17) underlying disease (Crohn’s disease, oral lichen planus, rheumatoid arthritis, scleroderma, Sjogren syndrome, dermato myositis, pemphigus vulgaris, polymyalgia rheumatica, systemic lupus erythematosus, oral cancer / squamous cell carcinoma), (18) CD4 cell count, (19) viral load, (20) prescribed antibiotic drug, (21) period in which the study was carried out, (22) type of effect estimate (relative risk, odds ratio, attributable risk/ excess risk, arcsine difference, standardized mean difference, weighted mean difference, hazard ratio), (23) value of effect estimate, (24) risk of bias assessment, (25) overall objectives of the study, (26) setting and/ or place of study, (27) additional information.

## Data Availability

All data are available in the manuscript and Supplementary files.

## References

[1] Vogel R, Smith-Palmer J, Valentine W (2013). Evaluating the health economic implications and cost-effectiveness of dental implants: a literature review. Int J Oral Maxillofac Implants.

[2] Esposito M, Thomsen P, Ericson LE, Lekholm U (1999). Histopathologic observations on early oral implant failures. Int J Oral Maxillofac Implants.

[3] Porter JA, von Fraunhofer JA (2005). Success or failure of dental implants? A literature review with treatment considerations. Gen Dent.

[4] Hwang D, Wang H-L. Medical contraindications to implant therapy: part I: absolute contraindications. Implant Dent. 2006;15(4):353–360. Implant Dentistry. 10.1097/01.id.0000247855.75691.03 17172952

[5] Kawai T, Akira S. Innate immune recognition of viral infection. Nat Immunol. 2006;7(2):131–137. Nature Publishing Group 10.1038/ni1303 16424890

[6] Midwood KS, Williams LV, Schwarzbauer JE (2004). Tissue repair and the dynamics of the extracellular matrix. Int J Biochem Cell Biol.

[7] Albrektsson T, Brånemark PI, Hansson HA, Lindström J (1981). Osseointegrated titanium implants. Requirements for ensuring a long-lasting, direct bone-to-implant anchorage in man. Acta Orthop Scand.

[8] Colnot C, Romero DM, Huang S, Rahman J, Currey JA, Nanci A (2007). Molecular analysis of healing at a bone-implant interface. J Dent Res.

[9] Harpaz R, Dahl RM, Dooling KL (2016). Prevalence of immunosuppression among US adults, 2013. JAMA.

[10] Jacobson DL, Gange SJ, Rose NR, Graham NM (1997). Epidemiology and estimated population burden of selected autoimmune diseases in the United States. Clin Immunol Immunopathol.

[11] Lerner A, Jeremias P, Matthias T (2016). The world incidence and prevalence of autoimmune diseases is increasing. Int J Celiac Dis.

[12] Mor G, Cardenas I. The immune system in pregnancy: a unique complexity. Am J Reprod Immunol. 2010;63(6):425–433. Blackwell Publishing Ltd. 10.1111/j.1600-0897.2010.00836.x PMC3025805 20367629

[13] Pedersen BK, Bruunsgaard H, Jensen M, Toft AD, Hansen H, Ostrowski K (1999). Exercise and the immune system--influence of nutrition and ageing. J Sci Med Sport.

[14] Gavazzi G, Krause K-H (2002). Ageing and infection. Lancet Infect Dis.

[15] Norman DC (2000). Fever in the elderly. Clin Infect Dis.

[16] Holzheimer RG, Mannick JA (2001). Surgical treatment: evidence-based and problem-oriented.

[17] Moher D, Liberati A, Tetzlaff J, Altman DG, The PRISMA Group. Preferred Reporting Items for Systematic Reviews and Meta-Analyses: the PRISMA statement. PLoS Med. 2009;6(7):e1000097. Public Library of Science. 10.1371/journal.pmed.1000097 PMC2707599 19621072

[18] Lefebvre C, Manheimer E, Glanville J, Higgins JP, Green S (2008). Searching for studies. Cochrane handbook for systematic reviews of interventions.

[19] Higgins JP, Green S, Higgins JP, Green S (2008). Guide to the contents of a Cochrane protocol and review. Cochrane handbook for systematic reviews of interventions.

[20] Green S, Higgins JP, Higgins JP, Green S (2008). Preparing a Cochrane review. Cochrane handbook for systematic reviews of interventions.

[21] Higgins JPT, Altman DG, Gøtzsche PC, Jüni P, Moher D, Oxman AD, et al. The Cochrane Collaboration’s tool for assessing risk of bias in randomised trials. BMJ 2011;343(oct18 2):d5928. British Medical Journal Publishing Group. 10.1136/bmj.d5928 PMC3196245 22008217

[22] Schwarzer G, Carpenter JR, Rücker G. Fixed effect and random effects meta-analysis. Meta-analysis with R. Cham: Springer International Publishing; 2015. 21–53. (Use R!).

[23] 2012 RCT, R Foundation for Statistical Computing Vienna, Austria. R: a language and environment for statistical computing. 2nd ed. R Foundation for Statistical Computing Vienna. 2010. Available from: http://www.R-project.org/. Cited 23 Mar 2013.

[24] news GSR, 2007. Meta: an R package for meta-analysis. bioconductorstatistiktu-dortmundde. https://bioconductor.statistik.tu-dortmund.de.

[25] Stevenson GC, Riano PC, Moretti AJ, Nichols CM, Engelmeier RL, Flaitz CM (2007). Short-term success of osseointegrated dental implants in HIV-positive individuals: a prospective study. JCDP.

[26] May MC, Andrews PN, Daher S, Reebye UN (2016). Prospective cohort study of dental implant success rate in patients with AIDS. Int J Implant Dent.

[27] Gherlone EF, Capparè P, Tecco S, Polizzi E, Pantaleo G, Gastaldi G (2015). A prospective longitudinal study on implant prosthetic rehabilitation in controlled HIV-positive patients with 1-year follow-up: the role of CD4+ level, smoking habits, and oral hygiene. Clin Implant Dent Relat Res.

[28] Gherlone EF, Capparè P, Tecco S, Polizzi E, Pantaleo G, Gastaldi G (2015). Implant prosthetic rehabilitation in controlled HIV-positive patients: a prospective longitudinal study with 1-year follow-up. Clin Implant Dent Relat Res.

[29] Gastaldi G, Vinci R, Francia MC, Bova F, Capparé P. Immediate fixed rehabilitation supported by axial and tilted implants of edentulous jaws: a prospective longitudinal study in HIV- positive patients. J Osseointegr. 2017;9(2):239–44.

[30] Maló P, de Araújo NM, Gonçalves Y, Lopes A (2015). Long-term outcome of implant rehabilitations in patients with systemic disorders and smoking habits: a retrospective clinical study. Clin Implant Dent Relat Res.

[31] Gay-Escoda C, Perez-Alvarez D, Camps-Font O, Figueiredo R (2016). Long-term outcomes of oral rehabilitation with dental implants in HIV-positive patients: a retrospective case series. Med Oral Patol Oral Cir Bucal.

[32] Baron M, Gritsch F, Hansy A-M, Haas R (2004). Implants in an HIV-positive patient: a case report. Int J Oral Maxillofac Implants.

[33] Achong RM, Shetty K, Arribas A, Block MS (2006). Implants in HIV-positive patients: 3 case reports. J Oral Maxillofac Surg.

[34] Kolhatkar S, Khalid S, Rolecki A, Bhola M, Winkler JR (2011). Immediate dental implant placement in HIV-positive patients receiving highly active antiretroviral therapy: a report of two cases and a review of the literature of implants placed in HIV-positive individuals. J Periodontol.

[35] Oliveira MA, Gallottini M, Pallos D, Maluf PSZ, Jablonka F, Ortega KL (2011). The success of endosseous implants in human immunodeficiency virus-positive patients receiving antiretroviral therapy a pilot study. J Am Dent Assoc.

[36] Castellanos-Cosano L, Núñez-Vázquez R-J, Segura-Egea J-J, Torres-Lagares D, Corcuera-Flores J-R, Machuca-Portillo G (2014). Protocol for oral implant rehabilitation in a hemophilic HIV-positive patient with type C hepatitis. Implant Dent.

[37] Vidal F, Vidal R, Bochnia J, de Souza RC, Gonçalves LS (2017). Dental implants and bone augmentation in HIV-infected patients under HAART: case report and review of the literature. Spec Care Dentist.

[38] Strietzel FP, Rothe S, Reichart PA, Schmidt-Westhausen A-M (2006). Implant-prosthetic treatment in HIV-infected patients receiving highly active antiretroviral therapy: report of cases. Int J Oral Maxillofac Implants.

[39] Alsaadi G, Quirynen M, Komárek A, van Steenberghe D (2008). Impact of local and systemic factors on the incidence of late oral implant loss. Clin Oral Implants Res.

[40] Moy PK, Medina D, Shetty V, Aghaloo TL (2005). Dental implant failure rates and associated risk factors. Int J Oral Maxillofac Implants.

[41] Ihara K, Goto M, Miyahara A, Toyota J, Katsuki T (1998). Multicenter experience with maxillary prostheses supported by Brånemark implants: a clinical report. Int J Oral Maxillofac Implants.

[42] Kovács AF (2001). Influence of chemotherapy on endosteal implant survival and success in oral cancer patients. Int J Oral Maxillofac Surg.

[43] Alsaadi G, Quirynen M, Michiles K, Teughels W, Komárek A, van Steenberghe D (2007). Impact of local and systemic factors on the incidence of failures up to abutment connection with modified surface oral implants. J Clin Periodontol.

[44] van Steenberghe D, Jacobs R, Desnyder M, Maffei G, Quirynen M (2002). The relative impact of local and endogenous patient-related factors on implant failure up to the abutment stage. Clin Oral Implants Res.

[45] Steiner M, Windchy A, Gould AR, Kushner GM, Weber R (1995). Effects of chemotherapy in patients with dental implants. J Oral Implantol.

[46] Sager RD, Theis RM (1990). Dental implants placed in a patient with multiple myeloma: report of case. J Am Dent Assoc.

[47] Bencharit S, Reside GJ, Howard-Williams EL (2010). Complex prosthodontic treatment with dental implants for a patient with polymyalgia rheumatica: a clinical report. Int J Oral Maxillofac Implants.

[48] Chochlidakis K, Ercoli C, Elad S (2016). Challenges in implant-supported dental treatment in patients with Sjogren’s syndrome: a case report and literature review. Quintessence Int.

[49] Ergun S, Katz J, Cifter ED, Koray M, Esen BA, Tanyeri H (2010). Implant-supported oral rehabilitation of a patient with systemic lupus erythematosus: case report and review of the literature. Quintessence Int.

[50] Krennmair G, Seemann R, Piehslinger E (2010). Dental implants in patients with rheumatoid arthritis: clinical outcome and peri-implant findings. J Clin Periodontol.

[51] Weinlander M, Krennmair G, Piehslinger E (2010). Implant prosthodontic rehabilitation of patients with rheumatic disorders: a case series report. Int J Prosthodont.

[52] Westhoff G, Doerner TDORACI, Cobarrubias K, Dietrich T, Zink A (2014). SAT0223 Dental implants are a treatment option in patients with Sjögren’s syndrome. Ann Rheum Dis.

[53] Zigdon H, Gutmacher Z, Teich S, Levin L (2011). Full-mouth rehabilitation using dental implants in a patient with scleroderma. Quintessence Int.

[54] de Mendonça IM, Finger Stadler A, Vale Nicolau G, Naval Machado MÂ, Soares de Lima AA, Compagnoni Martins M (2014). Management of Sjogren’s syndrome patient: a case report of prosthetic rehabilitation with 6-year follow-up. Case Rep Dent.

[55] Esposito SJ, Camisa C, Morgan M (2003). Implant retained overdentures for two patients with severe lichen planus: a clinical report. J Prosthet Dent.

[56] Marini E, Spink MJ, Messina AM (2013). Peri-implant primary squamous cell carcinoma: a case report with 5 years’ follow-up. J Oral Maxillofac Surg.

[57] Altin N, Ergun S, Katz J, Sancakli E, Koray M, Tanyeri H. Implant-supported oral rehabilitation of a patient with pemphigus vulgaris: a clinical report. J Prosthodont 2013;22(7):581–586. Wiley/Blackwell (10.1111). 10.1111/jopr.12050 23552022

[58] Binon PP (2005). Thirteen-year follow-up of a mandibular implant-supported fixed complete denture in a patient with Sjogren’s syndrome: a clinical report. J Prosthet Dent.

[59] Payne AG, Lownie JF, Van Der Linden WJ (1997). Implant-supported prostheses in patients with Sjögren’s syndrome: a clinical report on three patients. Int J Oral Maxillofac Implants.

[60] Ella B, Lasserre J-F, Blanchard J-P, Fricain JC (2011). A 4-year follow-up of two complete mandibular implant-supported removable prostheses in a patient with severe rheumatoid polyarthritis: case report. Int J Oral Maxillofac Implants.

[61] Alsaadi G, Quirynen M, Komárek A, van Steenberghe D (2007). Impact of local and systemic factors on the incidence of oral implant failures, up to abutment connection. J Clin Periodontol.

[62] Montebugnoli L, Venturi M, Cervellati F (2012). Bone response to submerged implants in organ transplant patients: a prospective controlled study. Int J Oral Maxillofac Implants.

[63] Montebugnoli L, Venturi M, Cervellati F, Servidio D, Vocale C, Pagan F (2014). Peri-implant response and microflora in organ transplant patients 1 year after prosthetic loading: a prospective controlled study. Clin Implant Dent Relat Res.

[64] Gu L, Wang Q, Yu Y-C (2011). Eleven dental implants placed in a liver transplantation patient: a case report and 5-year clinical evaluation. Chin Med J.

[65] Heckmann SM, Heckmann JG, Linke JJ, Hohenberger W, Mombelli A. Implant therapy following liver transplantation: clinical and microbiological results after 10 years. J Periodontol. 2004;75(6):909–13. 10.1902/jop.2004.75.6.909 15295960

[66] Nakagawa A, Shitara N, Ayukawa Y, Koyano K, Nishimura K (2014). Implant treatment followed by living donor lung transplant: a follow-up case report. J Prosthodont Res.

[67] Gu L, Yu Y-C (2011). Clinical outcome of dental implants placed in liver transplant recipients after 3 years: a case series. Transplant Proc.

[68] Campo J, Cano J, del Romero J, Hernando V, Rodríguez C, Bascones A. Oral complication risks after invasive and non-invasive dental procedures in HIV-positive patients. Oral Dis. 2007;13(1):110–116. Wiley/Blackwell (10.1111). 10.1111/j.1601-0825.2006.01262.x 17241440

[69] Kolhatkar S, Mason SA, Janic A, Bhola M, Haque S, Winkler JR (2012). Surgical crown lengthening in a population with human immunodeficiency virus: a retrospective analysis. J Periodontol.

[70] Lin CA, Takemoto S, Kandemir U, Kuo AC (2014). Mid-term outcomes in HIV-positive patients after primary total hip or knee arthroplasty. J Arthroplast.

[71] Ata-Ali J, Ata-Ali F, Di-Benedetto N, Bagan L, Bagan JV (2015). Does HIV infection have an impact upon dental implant osseointegration? A systematic review. Med Oral Patol Oral Cir Bucal.

[72] Ata-Ali J, Ata-Ali F (2014). Do antibiotics decrease implant failure and postoperative infections? A systematic review and meta-analysis. Int J Oral Maxillofac Surg.

[73] Keenan JR, Veitz-Keenan A. Antibiotic prophylaxis for dental implant placement? Evid Based Dent. 2015;16(2):52–53. Nature Publishing Group. 10.1038/sj.ebd.6401097 26114790

[74] Friedlaender GE, Tross RB, Doganis AC, Kirkwood JM, Baron R (1984). Effects of chemotherapeutic agents on bone. I. Short-term methotrexate and doxorubicin (adriamycin) treatment in a rat model. J Bone Joint Surg Am.

[75] Young DR, Virolainen P, Inoue N, Frassica FJ, Chao EYS. The short-term effects of cisplatin chemotherapy on bone turnover. J Bone Miner Res 1997;12(11):1874–1882. Wiley-Blackwell. 10.1359/jbmr.1997.12.11.1874 9383692

[76] Karr RA, Kramer DC, Toth BB (1992). Dental implants and chemotherapy complications. J Prosthet Dent.

[77] Chrcanovic BR, Albrektsson T, Wennerberg A (2016). Dental implants in patients receiving chemotherapy: a meta-analysis. Implant Dent.

[78] Smith DA, Germolec DR (1999). Introduction to immunology and autoimmunity. Environ Health Perspect.

[79] Cooper GS, Bynum MLK, Somers EC (2009). Recent insights in the epidemiology of autoimmune diseases: improved prevalence estimates and understanding of clustering of diseases. J Autoimmun.

[80] Weng X, Liu L, Barcellos LF, Allison JE, Herrinton LJ (2007). Clustering of inflammatory bowel disease with immune mediated diseases among members of a northern california-managed care organization. Am J Gastroenterol.

[81] Quirynen M, De Soete M, van Steenberghe D (2002). Infectious risks for oral implants: a review of the literature. Clin Oral Implants Res.

[82] Esposito M, Hirsch JM, Lekholm U, Thomsen P (1998). Biological factors contributing to failures of osseointegrated oral implants. (I). Success criteria and epidemiology. Eur J Oral Sci.

[83] Rustemeyer J, Bremerich A (2007). Necessity of surgical dental foci treatment prior to organ transplantation and heart valve replacement. Clin Oral Investig.

[84] Perdigão JPV, de Almeida PC, Rocha TDS, Mota MRL, Soares ECS, Alves APNN (2012). Postoperative bleeding after dental extraction in liver pretransplant patients. J Oral Maxillofac Surg.

