# Supplementary material for: Dental implants in immunocompromised patients: a systematic review and meta-analysis
Source: Int J Implant Dent. 2019 Nov 28;5:43. doi: 10.1186/s40729-019-0191-5 (PMC6881487; doi:10.1186/s40729-019-0191-5)
Supplement: Supplementary file 1 — Additional file 1. Detailed search history. The conducted search strategy used the databases: MEDLINE via the search interface OvidSP (keyword search) and Web of Science by Thompsen (all core databases: Science Citation Index, MEDLINE, Biosis). [file 40729_2019_191_MOESM1_ESM.docx]

**Detailed search history**

**1. Web of Science:**

(
("immune deficiency" OR "immun$deficiency" OR "immunocompetence" OR "imunosuppression" OR "immunocompromised" OR "immunologic deficiency syndrom*")

OR
"Immunotherapy"/
OR
("glucocorticoid*" OR "corticoid*")
OR
("chemotherap*" OR "drug therap*" OR "suppressive therap*" OR "antineoplastic therap*") OR

("cytostatic drug*" OR "cytostatic agent*" OR "cytostatic*" OR "antineoplastic agent*" OR "antineoplastic drug*" OR "prednisolone" OR "methotrexate" OR "mtx" OR "ametopterin" OR "dexamethasone")

OR
("cancer immunotherap*" OR "immunosupressiv* drug*" OR "immunosuppressiv* agent*")

OR

"antibody therap*"

OR

("calcineurin antagonist*" OR "calcineurin inhibitor*" OR "ciclosporin" OR "tacrolimus")

OR

("mTor inhibitor*" OR "everolimus" OR "sirolimus" OR "temsirolimus")

OR

("biopharmaceutical" OR "biologic* medical product*")

OR

("primary immunodeficienc*" OR "heriditery immunodeficienc*" OR "congenital immunodeficienc*" OR "autoimmune disease*“)

OR
"secondary immunodeficienc*"
OR
("hiv*" OR "aids" OR "haart" OR "antiretroviral" OR "hiv infection")

) AND

(
(("dental" OR "oral") NEAR/5 "implant*")

OR

((("osseointegrated" OR "endosseous") NEAR/3 "implant") AND ("dental" OR "oral"))

OR

((("overdenture*" OR "crown*" OR "bridge*" OR "prosthes$s" OR "restoration*" OR "blade") NEAR/5 ("dental" OR "oral")) AND "implant*")

)

**2. OVID**

(
("immune deficiency" OR "immun?deficiency" OR "immunocompetence" OR "imunosuppression" OR "immunocompromised" OR "immunologic deficiency syndrom*").mp.

OR
"Immunotherapy"/
OR
("glucocorticoid*" OR "corticoid*").mp.
OR
"Glucocorticoids"/
OR
("chemotherap*" OR "drug therap*" OR "suppressive therap*" OR "antineoplastic therap*").mp. OR

("cytostatic drug*" OR "cytostatic agent*" OR "cytostatic*" OR "antineoplastic agent*" OR "antineoplastic drug*" OR "prednisolone" OR "methotrexate" OR "mtx" OR "ametopterin" OR "dexamethasone").mp.

OR
("cancer immunotherap*" OR "immunosupressiv* drug*" OR "immunosuppressiv* agent*").mp. OR
"Drug Therapy"/
OR
"Antineoplastic Agents"/
OR
"antibody therap*".mp.
OR
("calcineurin antagonist*" OR "calcineurin inhibitor*" OR "ciclosporin" OR "tacrolimus").mp. OR
("mTor inhibitor*" OR "everolimus" OR "sirolimus" OR "temsirolimus").mp.
OR
("biopharmaceutical" OR "biologic* medical product*").mp.
OR

("primary immunodeficienc*" OR "heriditery immunodeficienc*" OR "congenital immunodeficienc*" OR "autoimmune disease*").mp.

OR

"Immunologic Deficiency Syndromes"/

OR

"Immune System Diseases"/

OR

"secondary immunodeficienc*".mp.

OR

("hiv*" OR "aids" OR "haart" OR "antiretroviral" OR "hiv infection").mp.

OR

"HIV"/

OR

"HIV Infections"/

OR

"Anti-HIV Agents"/ )

AND

(
(("dental" OR "oral") adj5 "implant*").mp.

OR

((("osseointegrated" OR "endosseous") adj3 "implant") AND ("dental" OR "oral")).mp.

OR

((("overdenture*" OR "crown*" OR "bridge*" OR "prosthes?s" OR "restoration*" OR "blade") adj5 ("dental" OR "oral")) AND "implant*").mp.

OR
"Dental Implants"/ OR
"Dental Implantation"/ OR

"Dental Prosthesis, Implant-Supported"/ )
