# Supplementary material for: Dental implants in immunocompromised patients: a systematic review and meta-analysis
Source: Int J Implant Dent. 2019 Nov 28;5:43. doi: 10.1186/s40729-019-0191-5 (PMC6881487; doi:10.1186/s40729-019-0191-5)
Supplement: Supplementary file 2 — Additional file 2. Detailed results table. (1) author, (2) publishing year, (3) study type, (4) medical condition (HIV infection, chemotherapy, transplantation, autoimmune disease, oral cancer / squamous cell carcinoma), (5) number of patients included in the study, (6) fraction of female and male patients, (7) age of the patients (median), (8) absolute number of placed implants, (9) absolute number of failed implants, (10) time of implantation after extraction, (11) time of loading, (11) maximum follow-up period, (12) survival rate of implants, (13) localization (mandible, maxilla), (14) generic therapy term (antiretroviral therapy, steroid containing medication, chemotherapy, immunosuppressive drugs), (15) applied drug, (16) duration of the applied therapy at the time of implantation, (17) underlying disease (Crohn’s disease, oral lichen planus, rheumatoid arthritis, scleroderma, Sjogren syndrome, dermato myositis, pemphigus vulgaris, polymyalgia rheumatica, systemic lupus erythematosus, oral cancer / squamous cell carcinoma), (18) CD4 cell count, (19) viral load, (20) prescribed antibiotic drug, (21) period in which the study was carried out, (22) type of effect estimate (relative risk, odds ratio, attributable risk/ excess risk, arcsine difference, standardized mean difference, weighted mean difference, hazard ratio), (23) value of effect estimate, (24) risk of bias assessment, (25) overall objectives of the study, (26) setting and/ or place of study, (27) additional information. [file 40729_2019_191_MOESM2_ESM.docx]

| **number** | **author** | **year** | **study_type** | **condition** | **patients** | **female** | **male** | **age** | **placed_implants** | **failed_implants** | **placement** | **loading** | **follow_up** | **survival_rate** | **localization** | **therapy_type** | **drug** | **duration** | **therapy_reason** | **CD4_cell_count** | **viral_load** | **antibiotics** | **total_period** | **ee-type** | **ee_value** | **rob** | **objective** | **remarks** | **location** |
| --- | --- | --- | --- | --- | --- | --- | --- | --- | --- | --- | --- | --- | --- | --- | --- | --- | --- | --- | --- | --- | --- | --- | --- | --- | --- | --- | --- | --- | --- |
| 002 | Achong | 2006 | case_a | hiv | 1 |  | 1 | 56 | 2 | 0 | 999 | 5 | 24 | 100,00% | mand | arv | HAART |  | hiv | 180 - 509 | 8 |  |  |  |  |  |  |  |  |
| 002 | Achong | 2006 | case_b | hiv | 1 |  | 1 | 45 | 2 | 0 | 999 | 6 | 24 | 100,00% | mand | arv | HAART |  | hiv | 202 - 468 | 4,5 |  |  |  |  |  |  |  |  |
| 002 | Achong | 2006 | case_c | hiv | 1 |  | 1 | 46 | 2 | 0 | 999 |  | 12 | 100,00% | mand | arv | HAART |  | hiv | 431 - 695 | 9,2 |  |  |  |  |  |  |  |  |
| 007 | Alsaadi | 2007 | retro | auto |  |  |  |  |  |  |  |  |  |  |  |  |  |  | crohn |  |  |  | 1983-2003 | or | 7,95 |  |  | 95% CI: (3.47, 18.24), P-value: 0.001 |  |
| 007 | Alsaadi | 2007 | retro | steroid |  |  |  |  |  |  |  |  |  |  |  |  |  |  |  |  |  |  | 1983-2003 | or | 1,25 |  |  |  |  |
| 008 | Alsaadi | 2008 | retro | auto | 6 |  |  |  | 28 | 0 |  |  | 24 | 100,00% | mand, max |  |  |  | rh |  |  |  |  |  |  |  | influence of systemic and local bone and intra-oral factors on the occurrence of implant loss from abutment connection up to 2 years |  | Department of Periodontology of the Catholic University Leuven |
| 008 | Alsaadi | 2008 | retro | auto | 2 |  |  |  | 9 | 3 |  |  | 24 | 66,67% | mand, max |  |  |  | crohn |  |  |  |  | or | 10,09 |  | influence of systemic and local bone and intra-oral factors on the occurrence of implant loss from abutment connection up to 2 years | 95% CI: (0.73, 139.79), P-value: 0.09 | Department of Periodontology of the Catholic University Leuven |
| 008 | Alsaadi | 2008 | retro | chemo | 3 |  |  |  | 10 | 0 |  |  | 24 | 100,00% | mand, max |  |  |  |  |  |  |  |  |  |  |  | influence of systemic and local bone and intra-oral factors on the occurrence of implant loss from abutment connection up to 2 years |  | Department of Periodontology of the Catholic University Leuven |
| 008 | Alsaadi | 2008 | retro | steroid | 5 |  |  |  | 17 | 0 |  |  | 14 | 100,00% | mand, max |  |  |  |  |  |  |  |  |  |  |  | influence of systemic and local bone and intra-oral factors on the occurrence of implant loss from abutment connection up to 2 years |  | Department of Periodontology of the Catholic University Leuven |
| 009 | Alsaadi | 2008 | pro | steroid |  |  |  |  | 4 | 0 |  |  | 6 | 100,00% | mand, max |  |  |  |  |  |  |  |  |  |  |  | the influence of systemic and local bone and intra-oral factors on the occurrence of early TiUnitet implant failures. | fisher: 1.00 GEE NA | Department of Periodontology of the University Hospital of the Catholic University of Leuven, |
| 009 | Alsaadi | 2008 | pro | chemo |  |  |  |  | 7 | 0 |  |  | 6 | 100,00% | mand, max |  |  |  |  |  |  |  |  |  |  |  | the influence of systemic and local bone and intra-oral factors on the occurrence of early TiUnitet implant failures. | fisher:1.00 GEE NA | Department of Periodontology of the University Hospital of the Catholic University of Leuven, |
| 009 | Alsaadi | 2008 | pro | auto |  |  |  |  | 12 | 1 |  |  | 6 | 91,67% | mand, max |  |  |  | crohn |  |  |  |  |  |  |  | the influence of systemic and local bone and intra-oral factors on the occurrence of early TiUnitet implant failures. | fisher: 0.21 GEE: 0.02n | Department of Periodontology of the University Hospital of the Catholic University of Leuven, |
| 009 | Alsaadi | 2008 | pro | auto |  |  |  |  | 14 | 1 |  |  | 6 | 92,86% | mand, max |  |  |  | rh |  |  |  |  |  |  |  | the influence of systemic and local bone and intra-oral factors on the occurrence of early TiUnitet implant failures. | fisher: 0.24 GEE: 0,22 | Department of Periodontology of the University Hospital of the Catholic University of Leuven, |
| 013 | Baron | 2004 | case | hiv | 1 | 1 |  | 42 | 12 | 0 | 2 | 7 | 24 | 100,00% | mand, max | arv | ART | 21 | hiv | 200 - 440 | <50 | clindamycin |  |  |  |  |  |  |  |
| 014 | Bencharit | 2010 | case_a | auto | 1 | 1 |  | 74 | 11 | 0 | 0 | 0 | 24 | 100,00% | mand, max | steroid | prednisolone | >12 | pr |  |  | amoxicillin |  |  |  |  |  |  |  |
| 014 | Bencharit | 2010 | case_a | auto | 1 | 1 |  | 74 | 1 | 0 | 0 | 2 | 19 | 100,00% | max | steroid | prednisolone | >12 | pr |  |  |  |  |  |  |  |  |  |  |
| 021 | Castellanos-Cosano | 2014 | case | hiv | 1 |  | 1 | 46 | 5 | 0 | 999 | 3 | 24 | 100,00% | mand | arv | HAART |  | hiv | 489 | 400 | amoxicillin, metamizole | |  |  |  |  |  |  |
| 023 | Chochlidakis | 2016 | case | auto | 1 | 1 |  | 71 | 6 | 0 | 0 | 0 | 14 | 100,00% | mand, max | steroid | triamcinolone |  | ss |  |  | amoxicillin |  |  |  |  |  |  |  |
| 038 | Ella | 2011 | case | auto | 1 | 1 |  |  | 2 | 0 |  |  | 48 | 100,00% | mand | is,steroid | mycophenolate mofetil, prednisone |  | rh |  |  |  |  |  |  |  |  |  |  |
| 040 | Ergun | 2010 | case | auto | 1 | 1 |  | 49 | 2 | 0 |  |  | 24 | 100,00% | mand | steroid | low-dose steroids |  | sle |  |  | amoxicillin |  |  |  |  |  |  |  |
| 044 | Gastaldi | 2017 | pro | hiv | 21 |  |  |  | 108 | 1, 5, 5 | 0, 999 | 0 | 24 | 95,37% | mand, max |  |  |  | hiv | 536.33 +/- 327.34 | | amoxicillin | 2013-2014 |  |  |  | success of implant prosthetic rehabilitation “All on four” in HIV positive patients | | San Luigi Center for Infectious Diseases, IRCCS San Raffaele Hospital, Milan, Italy |
| 045 | Gay-Escoda | 2016 | retro | hiv | 9 | 4 | 5 | 42 | 57 | 1 | 4 | 0, 3, 4 | 78 | 98,25% | mand, max | arv | ART |  | hiv | 242 - 1115 | <50 - 59 | amoxicillin |  |  |  |  |  |  |  |
| 047 | Gherlone | 2016 | pro | hiv | 68 | 22 | 46 | 55 | 194 | 15 | 999 | >1,64 | 24 | 92,11% | mand, max |  |  |  |  | >400 |  | amoxicillin |  |  |  |  |  | same patient collective as study 048 |  |
| 048 | Gherlone | 2016 | pro | hiv | 68 | 22 | 46 | 55 | 194 | 15 | 999 | >1,64 | 24 | 92,11% | mand, max |  |  |  |  | >400 |  | amoxicillin |  |  |  |  |  | same patient collective as study 047 |  |
| 051 | Gu | 2011 | case | trans | 1 | 1 |  | 45 | 11 | 0 | <1 | 0 | 60 | 100,00% | mand, max | is | tacrolimus | 24 | liver |  |  | moxifloxacin |  |  |  |  |  |  |  |
| 053 | Heckmann | 2004 | case | trans | 1 | 1 |  | 45 | 1 | 0 | 5 | 9 | 118 | 100,00% | max | is,steroid | prednisolone, tacrolimus, mycophenolate mofetil | 96 | liver |  |  |  |  |  |  |  |  |  |  |
| 057 | Kolhatkar | 2011 | case_a | hiv | 1 |  | 1 | 55 | 1 | 0 | 0 | 6 | 6 | 100,00% | mand | arv | HAART |  |  | 344 | 57 | azithromycin |  |  |  |  |  |  |  |
| 057 | Kolhatkar | 2011 | case_b | hiv | 1 |  | 1 | 48 | 1 | 0 | 0 | 11 |  | 100,00% | mand | arv | HAART |  |  | 379 | 32,551 | antibiotics |  |  |  |  |  |  |  |
| 057 | Kolhatkar | 2011 | case_b | hiv | 1 |  | 1 | 48 | 1 | 0 | 5 | 11 |  | 100,00% | mand | arv | HAART |  |  | 379 | 32,551 |  |  |  |  |  |  |  |  |
| 059 | Kovacs | 2001 | retro | chemo | 30 | 6 | 24 | 56 | 106 | 1, 1 |  |  | 120 | 99,10% | mand | chemo | cisplatin, carboplatin, 5-fluorouracil |  | scc |  |  | amoxicillin |  |  |  |  |  | Implantation after chemotherapy: Different data: 7.5 and 3 months; implant placement on average 10.5 months after the ablative operation; Fifteen of the 30 patients died during the observation period of 10 years. | |
| 059 | Kovacs | 2001 | retro | chemo | 17 | 4 | 13 | 55 | 54 | 1, 1 |  |  | 120 | 98,10% | mand |  |  |  | scc |  |  |  |  |  |  |  |  | implant placement on average 3 months after the ablative operation, nine of the 30 patients died during the observation period of 10 years | |
| 060 | Krennmair, G.; Seemann, R.; Piehslinger, E. | 2010 | retro | auto | 25 | 34 | 0 | 58.1 | 95 | 0 |  | 0 | 48 | 100,00% | mand, max | steroid |  |  | rh |  |  |  |  |  |  |  |  |  |  |
| 062 | Malo | 2016 | retro | auto | 36 |  |  |  | 212 | 17 | 12 |  | 120 | 72,00% | mand, max |  |  |  | rh |  |  |  | 1995-2012 |  |  |  |  | private clinic in Portugal (Malo Clinic Lisbon) |  |
| 062 | Malo | 2016 | retro | hiv | 5 |  |  |  | 40 | 2 | 12 |  | 60 | 95,00% | mand, max |  |  |  | hiv |  |  |  | 1995-2012 |  |  |  |  | private clinic in Portugal (Malo Clinic Lisbon) |  |
| 063 | May | 2016 | pro | hiv | 16 | 4 | 12 | 36 | 33 | 3 | 999 |  | 60 | 90,91% | mand, max | arv | HAART |  |  | <200 |  | not_applied |  |  |  |  |  | North Carolina community health center |  |
| 066 | Montebugnoli | 2012 | pro | trans | 10 | 2 | 8 | 58 | 20 | 0 | 12 | 3 | 3 | 100,00% | mand, max | is,steroid | cyclosporine, prednisone, sirolimus, tacrolimus | 24 | liver, heart |  |  |  |  |  |  |  |  | transplantations: eight livers, two hearts |  |
| 066 | Montebugnoli | 2012 | pro | control | 10 | 1 | 9 | 50 | 12 | 0 | 12 | 3 | 3 | 100,00% | mand, max |  |  |  |  |  |  |  |  |  |  |  |  |  |  |
| 067 | Montebugnoli | 2015 | pro | trans | 13 | 2 | 11 | 54 | 29 | 0 | 12 | 3 | 12 | 100,00% | mand, max | is | cyclosporine, tacrolimus |  | liver, heart |  |  | antibiotics |  |  |  |  |  | transplantation: eleven hearts, two livers |  |
| 067 | Montebugnoli | 2015 | pro | control | 13 | 5 | 8 | 53 | 28 | 0 | 12 | 3 | 12 | 100,00% | mand, max |  |  |  |  |  |  | antibiotics |  |  |  |  |  |  |  |
| 068 | Moy | 2005 | retro | chemo | 10 |  |  | 58 | 10 | 1 |  |  |  | 90,00% | mand, max |  |  |  |  |  |  |  |  | rr | 0,63 |  |  | Cl 0.08, 5.02 |  |
| 069 | Nakagwa | 2014 | case | trans | 1 | 2 |  | 45 | 1 | 0 | 5 | 9 | 118 | 100,00% | max |  |  | 0 | liver |  |  |  |  |  |  |  |  | 8 years after implant placement therapy started | |
| 074 | Oliveira | 2011 | pilot | hiv | 11 | 2 | 9 | 47 | 20 | 0 | 999 | 4 | 12 | 100,00% | mand | arv | HAART |  | hiv | 132 - 690 | <50 - 52 | amoxicillin |  |  |  |  |  | HIV-positive patients receiving protease inhibitor (PI)–based HAART | |
| 074 | Oliveira | 2011 | pilot | hiv | 14 | 3 | 11 | 46 | 20 | 0 | 999 | 4 | 12 | 100,00% | mand | arv | HAART |  | hiv | 129 - 1000 | <50 - 168 | amoxicillin |  |  |  |  |  | HIV- positive patients receiving nonnucleoside reverse transcriptase inhibitor–based HAART (without PI); | |
| 074 | Oliveira | 2011 | pilot | control | 15 |  |  |  | 20 | 0 | 999 | 4 | 12 | 100,00% | mand |  |  |  |  |  |  | amoxicillin |  |  |  |  |  |  |  |
| 084 | Rajnay | 1998 | case | hiv | 1 |  | 1 | 38 | 1 | 0 | 0 | 6 | 18 | 100,00% | mand | arv | ART |  | hiv | 150 - 200 | 600 - 35,000 | amoxicillin |  |  |  |  |  |  |  |
| 087 | Romanos | 2014 | case | hiv | 1 |  |  | 55 | 16 | 0 | 999 | 0 | 48 | 100,00% | mand, max | arv | ART |  | hiv | 479 |  | penicillin |  |  |  |  |  |  |  |
| 088 | Sager | 1990 | case | chemo | 1 |  | 1 |  | 4 | 0 |  |  | 18 | 100,00% | max | chemo,  steroid | melphalan, prednisone | 3 |  |  |  | cephalosporine |  |  |  |  |  |  |  |
| 093 | Shetty | 2005 | case | hiv | 1 |  | 1 | 47 | 8 | 0 | 999 | 6 | 36 | 100,00% | mand, max | arv | HAART |  | hiv | 170 - 459 | <50 | cefalexin |  |  |  |  |  |  |  |
| 101 | Steiner | 1995 | case | chemo | 1 | 1 |  | 67 | 2 | 0 | 999 | 4 | 3 | 100,00% | mand | chemo,  steroid | cytoxin, oncovin, prednisone | 0 |  |  |  |  |  |  |  |  |  | implantation after chemotherapy: chemotherapy started 3 weeks after implant placement (-0,69 months) | |
| 102 | Stevenson | 2007 | pro | hiv | 20 | 6 | 14 | 49 | 40 | 0 | 999 | 3 | 6 | 100,00% | mand | arv | HAART |  |  | 67 - 1247 | <50 - 113,576 | amoxicillin |  |  |  |  |  |  |  |
| 102 | Stevenson | 2007 | pro | control | 9 |  |  |  | 18 | 0 | 999 | 3 | 6 | 100,00% | mand |  |  |  |  |  |  |  |  |  |  |  |  |  |  |
| 103 | Strietzel | 2006 | case_a | hiv | 1 |  | 1 | 64 | 1 | 0 | 999 | 6 | 30 | 100,00% | mand | arv | HAART | 36 |  | 408 | <50 | not_applied |  |  |  |  |  |  |  |
| 103 | Strietzel | 2006 | case_a | hiv | 1 |  | 1 | 64 | 4 | 1 | 999 | 4 | 37 | 75,00% | mand | arv | HAART | 36 |  | 408 | <50 | not_applied |  |  |  |  |  |  |  |
| 103 | Strietzel | 2006 | case_b | hiv | 1 |  | 1 | 38 | 2 | 0 | 999 | 4 | 28 | 100,00% | mand | arv | HAART | 24 |  | 800 | <50 | not_applied |  |  |  |  |  |  |  |
| 103 | Strietzel | 2006 | case_c | hiv | 1 | 1 |  | 49 | 4 | 0 | 999 | 4 | 25 | 100,00% | mand | arv | HAART | 48 |  | 576 | <50 | not_applied |  |  |  |  |  |  |  |
| 115 | van Steenberghe | 2002 | pro | auto |  |  |  | 50 |  |  |  |  | 6 | 46,50% | mand, max |  |  |  | crohn |  |  |  |  |  |  |  |  | combined risk factors |  |
| 115 | van Steenberghe | 2002 | pro | chemo |  |  |  | 50 |  |  |  |  | 6 | 89,00% | mand, max |  |  |  |  |  |  |  |  |  |  |  |  |  |  |
| 121 | Vidal | 2016 | case_a | hiv | 1 | 1 |  | 56 | 1 | 0 | 4 | 4 | 122 | 100,00% | mand | arv | HAART | 60 |  | 1,163 | <50 |  |  |  |  |  |  |  |  |
| 121 | Vidal | 2016 | case_b | hiv | 1 |  | 1 | 46 | 1 | 0 | 0 | 6 | 122 | 100,00% | max | arv | HAART | 132 |  | 1,598 | <50 | amoxicillin |  |  |  |  |  |  |  |
| 121 | Vidal | 2016 | case_c | hiv | 1 |  | 1 | 62 | 2 | 0 | 999 | 8 | 61 | 100,00% | max | arv | HAART | 84 |  | 1,45 | <50 | amoxicillin |  |  |  |  |  | 13 years re-evaluation of the #36 implant, der war schon drin, sinus floor elevation | |
| 124 | Weinlander | 2010 | retro | auto | 4 | 4 |  | 56 | 21 | 0 |  |  | 46 | 100,00% | mand, max | steroid | corticosteroids |  | rh,ss |  |  |  |  |  |  |  |  |  |  |
| 124 | Weinlander | 2010 | retro | auto | 1 | 1 |  | 56 | 2 | 0 |  |  | 46 | 100,00% | max | steroid | corticosteroids |  | rh,dm |  |  |  |  |  |  |  |  |  |  |
| 124 | Weinlander | 2010 | retro | auto | 1 | 1 |  | 56 | 6 | 0 |  |  | 46 | 100,00% | mand | steroid | corticosteroids |  | sc |  |  |  |  |  |  |  |  |  |  |
| 124 | Weinlander | 2010 | retro | auto | 16 | 16 |  | 56 | 60 | 0 |  |  | 46 | 100,00% | mand, max | steroid | corticosteroids |  | rh |  |  |  |  |  |  |  |  |  |  |
| 125 | Westhoff | 2012 | retro | auto | 20 |  |  | 58 | 60 | 4 |  |  |  | 93,00% |  | steroid | glucocorticoids |  | ss |  |  |  |  |  |  |  |  | 7 page oral health questionnaire |  |
| 135 | Zigdon | 2011 | case | auto | 1 | 1 |  | 45 | 12 | 0 | 4 | 9 | 36 | 100,00% | mand, max | steroid | glucocorticoids |  | sc |  |  | antibiotics |  |  |  |  |  |  |  |
| 136 | Gu | 2011 | case | trans | 13 | 3 | 10 | 58 | 45 | 0 | >2 | 6 | 36 | 100,00% | mand, max | is | tacrolimus, mycophenolate mofetil, CsA |  | liver |  |  | amoxicillin,  clavulanate  potassium | | |  |  |  |  |  |
| 143 | de Mendonça | 2014 | case | auto | 1 | 1 |  | 58 | 2 | 0 |  | 4 | 72 | 100,00% | max | steroid | prednisone |  | rh,ss |  |  | amoxicillin |  |  |  |  |  |  |  |
| 147 | Esposito | 2003 | case_b | auto | 1 | 1 |  | 78 | 2 | 0 |  | 3 | 18 | 100,00% | mand | steroid | steroids |  | olp,scc |  |  |  |  |  |  |  |  |  |  |
| 147 | Esposito | 2003 | case_a | auto | 1 | 1 |  | 72 | 2 | 0 |  | 3 | 18 | 100,00% | mand | steroid | steroids |  | olp,ss |  |  |  |  |  |  |  |  |  |  |
| 148 | Marini | 2013 | case | auto | 1 | 1 |  | 51 | 2 | 1 | 999 | 3 | 60 | 50,00% | mand | steroid | prednisone |  | olp,scc |  |  |  |  |  |  |  |  | scc |  |
| 149 | Altin | 2013 | case | auto | 1 | 1 |  | 70 | 2 | 0 | 999 | 3 | 32 | 100,00% | mand | steroid | deflazocort (steroid) |  | pv |  |  | amoxicillin |  |  |  |  |  |  |  |
| 156 | Binon | 2005 | case | auto | 1 |  | 1 | 67 | 6 | 0 | 0 | 4 | 156 | 100,00% | mand | steroid | prednisone |  | rh,ss |  |  |  |  |  |  |  |  |  |  |
| 158 | Payne | 1997 | case | auto | 1 | 1 |  | 38 | 6 | 0 |  |  | 96 | 100,00% | mand, max | steroid | cortisone |  | rh,ss |  |  |  |  |  |  |  |  |  |  |
| 159 | Ihara | 1998 | retro | chemo | 1 |  |  | 64 | 5 | 0 |  |  | 28 | 100,00% |  | chemo | chemo |  | scc, others |  |  |  |  |  |  |  |  |  |  |
|  |  |  |  |  |  |  |  |  |  |  |  |  |  |  |  |  |  |  |  |  |  |  |  |  |  |  |  |  |  |

| Legende: |  | |  | |
| --- | --- | --- | --- | --- |
| color | case reports/ case series | | | |
| color | retrospective / prospective studies (without controll) | | | |
| color | conrolled studies | | | |
| number |  | | numerical listing accordingt to the search record | |
| author |  | | first author of the study accordingt to the search record | |
| year |  | | publishing year accordingt to the search record | |
| study type |  | | case report = case; retrospective study = retro, pilot study = pilot, | |
| condition |  | | medical condition: hiv= humane immunodeficiency virus, chemo=chemotherapy, trans=transplantation, auto=autoimmune disease, scc=oral cancer / squamos cell carcinoma | |
| patients |  | | number of patients | |
| female |  | | fraction of female patients | |
| male |  | | fraction of male patients | |
| age |  | | age of the patients (median) | |
| placed_implants | | absolute number of placed implants | |  |
| failed_implants | | absolute number of failed implants | |  |
| placement |  | | # = implant placement given in months after extraction, 999 = no extraction prior implant placement, 0=immediately | |
| loading |  | | # = implant loading given in months after implant placement | |
| follow_up |  | | maximum follow-up period given in month | |
| survival_rate | | given in percentage | |  |
| localization |  | | mand = mandible, max = maxilla | |
| therapy_type | | generic therapy term: arv=antiretroviral therapy, steroid=steroid containing medication, chemo=chemotherapy, is=immunosupressive drugs | |  |
| drug |  | | applied drug to treat the medical condition | |
| duration |  | | duration of the applied therapy at the time of implantation | |
| therapy_reason | | underlying disease: crohn=crohn's diesease, olp=oral lichen planus, rh=rheumatoid arthritis, sc=scleroderma, ss=sjögren syndrome, dm=dermato myositis, pv=pemphigus vulgaris, pr=polymyalgia rheumatica, sle=systemic lupus erythematosus, scc=oral cancer / squamos cell carcinoma | |  |
|  |  | |  | |
| CD4_cell_count | | CD4 cell count: <200 very_low, 200-500 low, >500 normal | |  |
| viral_load |  | | viral load: <50 copies = undedectable, 50-10000 =low, 10000-100000 = normal, >100000 = high) | |
| antibiotics |  | | name of the antibiotic drug | |
|  |  | |  | |
| total_period | | period in which the study was carried out | |  |
|  |  | |  | |
| ee_type |  | | type of effect estimate: rr = relative risk, or = odds ratio, ad = attributable risk / excess risk, as = Arcussinus-Differenz, sdm = standardisierte mittlere Differenz, wdm = gewichtete mittlere Differenz, hr = Hazard-Ratio | |
| ee_value |  | | value of effect estimate | |
| rob |  | | risk of bias assessment | |
| objectives |  | | overall objectives of the study | |
| remarks |  | | additional information | |
| location |  | | setting and/or place of study | |
|  |  | |  | |
| blank field |  | | no information given | |
